# Low-cost diagnostic test for susceptible and drug-resistant tuberculosis in rural Malawi

**DOI:** 10.4102/ajlm.v7i1.690

**Published:** 2018-06-04

**Authors:** Annie Zhang, Enoch Jumbe, Robert Krysiak, Sabeen Sidiki, Holden V. Kelley, Elly K. Chemey, Chancy Kamba, Victor Mwapasa, Juan I. García, Alison Norris, Xueliang J. Pan, Carlton Evans, Shu-Hua Wang, Jesse J. Kwiek, Jordi B. Torrelles

**Affiliations:** 1 Department of Microbiology, College of Arts and Sciences, The Ohio State University, Columbus, Ohio, United States; 2 Child Legacy International, Msundwe, Lilongwe, Malawi; 3 Department of Infectious Diseases, School of Medicine, University of North Carolina Project, Tidziwe Centre, Lilongwe, Malawi; 4 Department of Microbial Infection and Immunity, College of Medicine, The Ohio State University, Columbus, Ohio, United States; 5 District Tuberculosis Control Office, Ministry of Health, Lilongwe, Malawi; 6 Department of Community Health, College of Medicine, Blantyre, Malawi; 7 Department of Pediatrics, Obstetrics, Gynecology and Preventive Medicine, Autonomous University of Barcelona, Barcelona, Spain; 8 College of Public Health, The Ohio State University, Columbus, Ohio, United States; 9 Center for Biostatistics, College of Medicine, The Ohio State University, Columbus, Ohio, United States; 10 The Wellcome Centre for Clinical Tropical Medicine, Imperial College of London, London, United Kingdom; 11 Department of Microbiology, Cayetano Heredia University, Lima, Peru; 12 Division of Infectious Diseases, Department of Internal Medicine, College of Medicine, The Ohio State University, Columbus, Ohio, United States

## Abstract

**Background:**

Rural settings where molecular tuberculosis diagnostics are not currently available need easy-to-use tests that do not require additional processing or equipment. While acid-fast bacilli (AFB) smear is the most common and often only tuberculosis diagnosis test performed in rural settings, it is labour intensive, has less-than-ideal sensitivity, and cannot assess tuberculosis drug susceptibility patterns.

**Objective:**

The objective of this study was to determine the feasibility of a multidrug-resistant (MDR) or extensively drug-resistant (XDR)-tuberculosis coloured agar-based culture test (tuberculosis CX-test), which can detect *Mycobacterium tuberculosis* growth and evaluate for drug susceptibility to isoniazid, rifampicin and a fluoroquinolone (i.e. ciprofloxacin) in approximately 14 days.

**Method:**

In this study, 101 participants were enrolled who presented to a rural health clinic in central Malawi. They were suspected of having active pulmonary tuberculosis. Participants provided demographic and clinical data and submitted sputum samples for tuberculosis testing using the AFB smear and tuberculosis CX-test.

**Results:**

The results showed a high level of concordance between the AFB smear (12 positive) and tuberculosis CX-test (13 positive); only one sample presented discordant results, with the molecular GeneXpert MTB/RIF^®^ test confirming the tuberculosis CX-test results. The average time to a positive tuberculosis CX-test was 10 days. Of the positive samples, the tuberculosis CX-test detected no cases of drug resistance, which was later confirmed by the GeneXpert MTB/RIF^®^.

**Conclusion:**

These findings demonstrate that the tuberculosis CX-test could be a reliable low-cost diagnostic method for active pulmonary tuberculosis in high tuberculosis burden rural areas.

## Introduction

In 2017, the World Health Organization (WHO) estimated that 4000 people die of tuberculosis every day. Malawi is among the 20 countries with a WHO-defined ‘high’ tuberculosis and HIV burden: the country reported 15 737 new and relapsed cases in 2015.^1^ Of these cases, only 6% were tested with rapid tuberculosis diagnostics at the time of diagnosis (by GeneXpert MTB/RIF^®^, Cepheid, Sunnyvale, California, United States).^2^ Most, 75%, were diagnosed as pulmonary tuberculosis by clinical symptoms; 58% of these had confirmatory culture and only 47% were provided with tuberculosis treatment. The recently-reported tuberculosis incidence rate in Malawi is 193 per 100 000 people per year, with 53% of cases occurring in people that are also HIV-positive.^1^ WHO data for Malawi estimates that 0.75% of new cases and 6.4% of previously-treated cases are multidrug-resistant (MDR)-tuberculosis^1^; however, real numbers may be higher due to current limited drug susceptibility testing in the country.

With the adoption of the ‘Sustainable Development Goals’ and the ‘End TB Strategy’ in late 2015, worldwide efforts to end the global tuberculosis epidemic are ambitious and require new advancements in tuberculosis diagnostics. While acid-fast bacilli (AFB) smear is the most common tuberculosis diagnostic method in Malawi and many other low-income and high tuberculosis-burden countries around the globe, it is labour intensive, has less-than-ideal sensitivity, and cannot be used to assess tuberculosis drug susceptibility. Access to tuberculosis diagnostics has improved in recent years but still remains limited. In Malawi, there are approximately two smear and microscopy facilities per 100 000 people and one laboratory capable of performing tuberculosis drug susceptibility tests per 10 million people.^3^ Culture-based diagnostic methods remain the gold standard for drug susceptibility testing, but can take up to 86 days to yield results.^4^ In 2010, the WHO recommended GeneXpert MTB/RIF^®^ as an initial tuberculosis diagnostic test. The GeneXpert MTB/RIF^®^ is a polymerase chain reaction-based test that takes less than two hours to perform and simultaneously detect *Mycobacterium tuberculosis* and rifampicin resistance in the tested sample. In this test, rifampicin resistance is used as a surrogate marker for MDR-tuberculosis, which is defined as resistance to both isoniazid and rifampicin. Despite its sensitivity and utility, the GeneXpert MTB/RIF^®^ implementation in high tuberculosis burden areas is cost prohibitive (approximately $18 per sample in Malawi), requires expensive instrumentation with weekly maintenance and monthly calibration, a sustained power source, and laboratory technicians with specialised training.^5^ These drawbacks render it currently inaccessible to most areas in high tuberculosis-burden countries. There is a need for a simple, inexpensive tuberculosis diagnostic test that can be performed in rural health facilities, where access to molecular tuberculosis diagnostics may not be feasible.

The MDR/XDR-tuberculosis coloured agar-based test (tuberculosis CX-test) is a non-commercial, thin-layer agar-based tuberculosis culture method capable of simultaneously detecting *M. tuberculosis* and tuberculosis drug susceptibility in approximately 14 days. The tuberculosis CX-test contains four quadrants: one quadrant detects *M. tuberculosis* growth and the other three quadrants detect resistance to isoniazid, rifampicin and a fluoroquinolone (i.e. ciprofloxacin). The tuberculosis CX-test is simple to use: expectorated sputum is mixed with disinfectant, the mixture is cultured onto the tuberculosis CX-test, and colonies are enumerated after incubation. In a study completed in a research laboratory setting using 197 archived *M. tuberculosis* clinical isolates, the tuberculosis CX-test detected drug resistance with 98% sensitivity for isoniazid, rifampicin, and ciprofloxacin and 99% for MDR-tuberculosis, compared to drug susceptibility testing results using a liquid culture method.^6^ Specificities reported for isoniazid were 100% (95% CI 82–100), 88% (95% CI 69–97) for rifampicin, 91% (95% CI 83–96) ciprofloxacin and 90% (95% CI 74–98) for MDR-tuberculosis.^6,7^ A systematic review of three studies assessing the thin-layer agar-based assay technique found a pooled sensitivity of 100% (95% CI 97–100) and pooled specificity of 100% (95% CI 99–100) for the detection of rifampicin; and a pooled sensitivity of 100% (95% CI 91–100) and pooled specificity of 100% (95% CI 99–100) for the detection of isoniazid.^8,9,10,11^ The tuberculosis CX-test has also been shown to be highly specific in identifying *M. tuberculosis* from atypical mycobacteria.^12^ Although the tuberculosis CX-test has been shown to be accurate in research laboratories, its performance in the field and in clinical settings in high tuberculosis-burden areas has yet to be characterised.

In this study, we sought to determine the feasibility of the tuberculosis CX-test to diagnose active pulmonary tuberculosis and patterns of tuberculosis drug susceptibility to isoniazid, rifampicin, and ciprofloxacin in a rural Malawian health clinic using direct sputum specimens, where currently only AFB smear is performed and no routine cultures are sent for confirmation or drug susceptibility testing.

## Methods

### Ethical considerations

Institutional review board approval was obtained from The Ohio State University (study number 2014H0381) and the Malawi College of Medicine Research and Ethics Committee (study number P.09/14/1627). All participants were adults and were enrolled in the study using a written consent form. Tuberculosis CX-test results were for research only and were not used for diagnosis and they did not influence treatment outcomes. Following the Malawian Ministry of Health recommendations, GeneXpert MTB/RIF^®^ positive samples were to be retested by the Malawian National Tuberculosis Control Program before patients were notified.

### Study population

Participants were recruited from the Child Legacy International-McGuire Wellness Center in Msundwe, Malawi. Child Legacy International serves a catchment area of 68 rural villages (~18 000 people) in the central region of Malawi.^13,14,15,16,17^ Participants were limited to patients suspected of having active pulmonary tuberculosis disease based on clinical symptoms (i.e. fever, chills, night sweats, shortness of breath, chest pain, cough ≥ 2 weeks, loss of weight, fatigue), who were ≥ 18 years of age, capable of providing informed written consent, and able to provide sputum. Participants answered a survey regarding demographics (i.e. age, sex) and clinical history (i.e. HIV status, tuberculosis clinical symptoms, previous diagnosis and treatment). HIV status and testing were verified by Health Passport or offered on-site to all participants, along with pre- and post-HIV counselling; HIV testing was not required for participation in this study. Participants were recruited over the course of 11 months during the period of November 2015 to October 2016.

### Tuberculosis CX-test preparation

The tuberculosis CX-tests were prepared according to published methods as previously described^6^ in different batches in research laboratories at The Ohio State University in the United States. Tuberculosis CX-test quality control per each batch was confirmed in an Ohio State University Biosafety Level 3 laboratory using verified susceptible, mono-isoniazid resistant, mono-rifampicin resistant and MDR *M. tuberculosis* clinical isolates provided by the Ohio Department of Health State Laboratory. Upon tuberculosis CX-test quality control confirmation, tuberculosis CX-test batches were transported to Malawi and properly stored at 4 °C until used within four months of being made.

### Tuberculosis CX-testing

Sputum samples were collected, numerically coded (de-identified) and equally divided by pipetting for (1) AFB smear, (2) tuberculosis CX-test, and (3) GeneXpert MTB/RIF^®^ testing (in the case of discordant results). Sputa for AFB smear and tuberculosis CX-tests were processed immediately after collection, whereas samples for GeneXpert MTB/RIF^®^ testing were stored at −20 °C until used.

For the AFB smear, standard procedures dictated by the Malawi Ministry of Health were followed for sputum collection, Ziehl-Neelsen technique for AFB staining, microscopy, and smear grading.^18^ During Ziehl-Neelsen staining, sputum was applied to a slide and heat fixed. The slide was submerged into carbol fuchsin, heated to dry, and rinsed with water. Next, slides were submerged in a 3% solution of hydrochloric acid (de-staining step), briefly washed with water, and then counterstained with methylene blue.^19^

**FIGURE 1:**
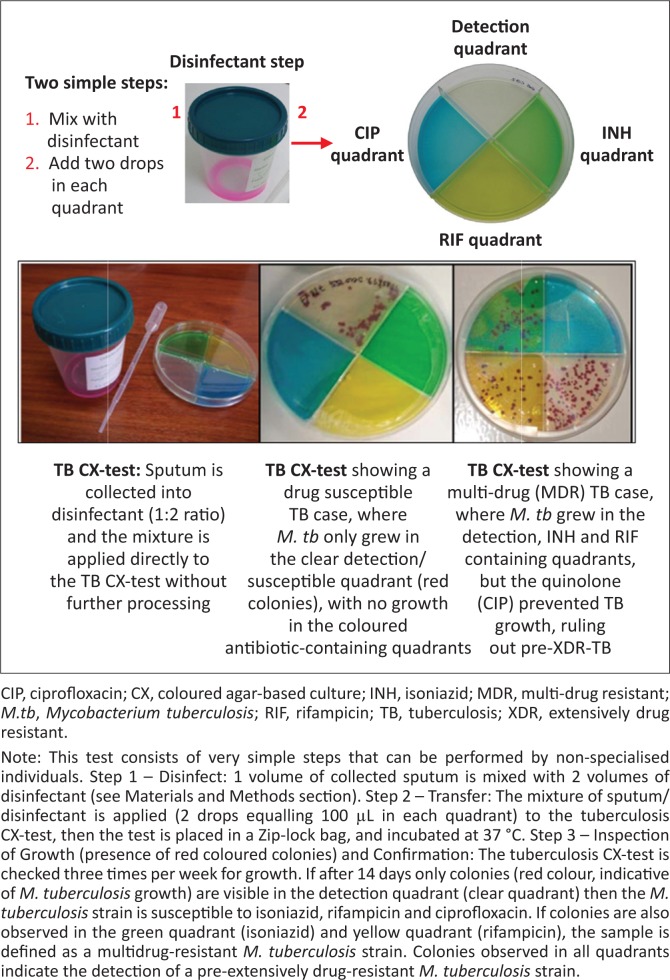
The tuberculosis CX-test.

For the tuberculosis CX-test (Figure 1), one-third of the collected sputum was added to a 50 mL tube containing twice the volume of disinfectant (stock solution: 2 g tri-sodium phosphate, 0.05 g ammonium sulphate, 0.005 g magnesium sulphate, 0.0025 g ferric ammonium citrate, 10 mL sterile water, and 0.01 mL red food coloring, all mixed by manual shaking). Two drops of the sputum/disinfectant mixture (1:2, v/v) were then plated directly onto each quadrant of the tuberculosis CX-test.^6^ The tuberculosis CX-test was then incubated at 37 °C for 28–42 days and checked three times per week for bacterial growth. Colonies from each quadrant were counted during each check. The presence of *M. tuberculosis* was microscopically defined as the presence of the typical rough colony on the detection quadrant, using a low-resolution (20X) microscope (clear quadrant). Drug resistance was defined by the detection of growth on each of the specific quadrants: isoniazid (yellow quadrant), rifampicin (green quadrant), and ciprofloxacin (blue quadrant). The drug concentration in each quadrant was as follows: isoniazid (0.2 µg/mL), rifampicin (1 µg/mL) and ciprofloxacin (2 µg/mL). As a precautionary measure, all tuberculosis CX-tests were kept in the incubator for an additional seven days before being discarded.

For discordant results between the AFB smear and the tuberculosis CX-test, coded sputum samples stored at −20 °C were transported to Lilongwe and tested with GeneXpert MTB/RIF^®^ by the University of North Carolina Project at Lilongwe, as described previously.^1^ Moreover, approximately an additional 10% of samples tested by AFB smear and the tuberculosis CX-test were selected randomly and also analysed by GeneXpert MTB/RIF^®^ to confirm the obtained results.

### Statistical analysis

Descriptive data regarding participant demographics and clinical characteristics were collected and summary statistics were compiled. The Wilcoxon Rank-Sum test was used to test age association with tuberculosis diagnosis. Fisher’s exact test was used to identify clinical characteristics significantly associated with a positive tuberculosis diagnosis. Data analysis was performed using JMP software (version 11; SAS [https://www.jmp.com/en_us/home.html]).

## Results

A total of 101 participants were enrolled in the study. Of these, five participants were excluded due to tuberculosis CX-plate contamination or the plate drying out. The average participant age was 47 years (SD = 17) (Table 1). A large percentage of participants had been already tested for HIV (97%) at enrolment. Of these, 10 (10.4%) stated they were HIV-positive, 82 (85.4%) stated they were HIV-negative, while the remaining 4 (4.2%) did not know their status or had never been tested. The majority (88.5%) had never been tested previously for tuberculosis, while eleven (11.5%) had previous tuberculosis testing but of these only eight (8.3%) were diagnosed with active pulmonary tuberculosis. Of the 10 HIV-positive participants, only two (20%) reported they were taking antiretroviral treatment. No participant reported taking tuberculosis medication at the time of enrolment. The participants’ most commonly-reported symptoms included dry and persistent cough (97%), weight loss (71%), fatigue (63%), night sweats (55%), fever (54%), chills (39%), and bloody sputum (30%). None of these symptoms was found to be significantly associated with a positive tuberculosis diagnosis.

**TABLE 1:** Participant demographics and clinical characteristics.

Demographic	*N*†	*N*	%
**Age**	**96**	**47.0 ± 17.4**‡ **(Range: 19–90)**	
18–29	-	17	18
30–39	-	21	22
40–49	-	16	17
50–59	-	13	13
60–69	-	16	17
70–79	-	9	9
80–89	-	3	3
90–99	-	1	1
**Tested for HIV**	96	-	-
Yes	-	93	97
No	-	3	3
**HIV status**	96	-	-
Positive	-	10	10
Negative	-	82	85
Don’t know	-	2	2
Never tested	-	2	2
**Taking ART**	10	-	-
Yes	-	2	20
No	-	8	80
**Previously tested for TB**	96	-	-
Yes	-	11	11
No	-	85	89
**Previously diagnosed with TB**	11	-	-
Yes	-	8	73
No	-	3	27
**Taking TB medication**	96	-	-
Yes	-	0	-
No	-	96	100
**Symptoms§**	96	-	-
Cough	-	93	97
Blood in sputum	-	29	30
Fever	-	52	54
Chills	-	37	39
Night sweats	-	53	55
Weight loss	-	68	71
Fatigue	-	60	63

ART, antiretroviral treatment; TB, tuberculosis.

† , Some questions were not answered by participants and have been excluded from this table.

‡ , Age mean ± SD.

§ , Some patients presented multiple symptoms.

**FIGURE 2:**
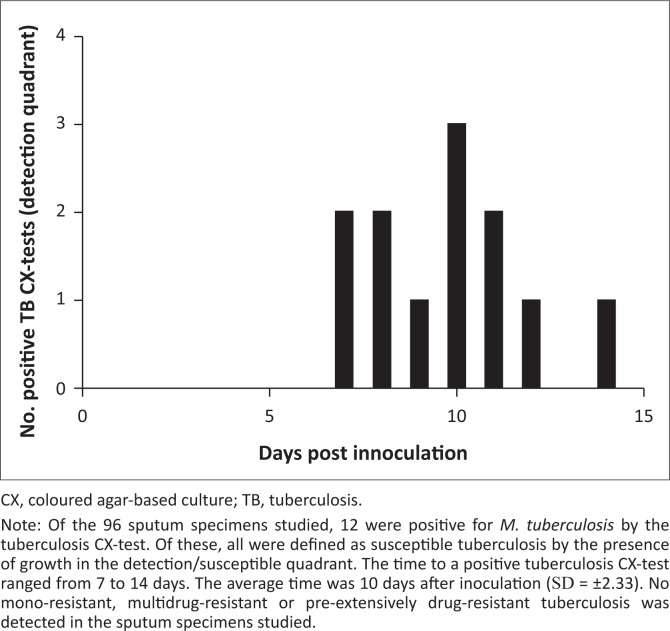
Time to positive tuberculosis CX-test.

Of the 96 participants, 12 (12.5%) were positive by both AFB smear and the tuberculosis CX-test, and 83 (86.5%) were negative by both AFB smear and the tuberculosis CX-test (Table 2). One sample was found to have discordant results between AFB smear (negative) and the tuberculosis CX-test (positive). In this case, the GeneXpert MTB/RIF^®^ revealed a positive result for the presence of *M. tuberculosis* in sputum, in agreement with the tuberculosis CX-test. The tuberculosis CX-test was also comparable to the AFB smear (99% agreement on the diagnosis results), and positively identified a tuberculosis diagnosis, while AFB smear yielded a single false negative result. The time to obtain a positive tuberculosis CX-test result ranged between 7 and 14 days (mean = 10, SD = ±2.33) (Figure 2). The tuberculosis CX-test detected no cases of drug resistance; however, late growth was observed in the antibiotic quadrants of three plates. Late growth is defined as growth in any of the antibiotic quadrants that takes place after growth in the detection quadrant is observed. This growth is not indicative of drug resistance. The GeneXpert MTB/RIF^®^ testing of nine positive samples that were sent for verification confirmed the presence of *M. tuberculosis* and no rifampicin drug resistance, including the three samples that contained late growth in the rifampicin quadrant. Among patients with a positive tuberculosis diagnosis, three (23%) stated they were also HIV-positive. Younger age (18 to 39-years old) was significantly associated with a positive tuberculosis diagnosis (*Z* = −2.2, *p* < 0.03).

**TABLE 2:** Contingency table of acid-fast bacilli smear microscopy vs. tuberculosis CX-test.

TB CX-test	AFB staining (+)	AFB staining (−)	Totals
TB CX-test(+)	12	1*	13
TB CX-test(−)	0	83	83
**Totals**	**12**	**84**	**96**

AFB, acid-fast bacilli; CX, coloured agar-based culture; TB, tuberculosis.

* , GeneXpert MTB/RIF^®^ confirmed that the specimen was *M. tuberculosis* positive.

## Discussion

Our findings demonstrate that the tuberculosis CX-test can be implemented in rural health clinics in a low-income and high tuberculosis-burden setting, with diagnosis sensitivity comparable to AFB smear. The sensitivity of AFB staining is dependent on the presence of high bacterial load in the patient’s sputum and the technical skills of the microscopist. One study estimated that AFB smear microscopy fails to detect *M. tuberculosis* in a third of patients who are later diagnosed by culture, and that AFB smear microscopy is particularly insensitive in high-risk tuberculosis populations including children and HIV-positive individuals.^5^ Moreover, in areas with high incidence of nontuberculous mycobacteria infections, the AFB smear cannot distinguish clearly between nontuberculous mycobacteria and *M. tuberculosis*. However, studies that used the tuberculosis CX-test demonstrated that this test is able to differentiate *M. tuberculosis* from nontuberculous mycobacteria infections with > 99.6% specificity.^6^ In this study, there was high concordance between AFB staining and the tuberculosis CX-test, with the latter offering higher sensitivity over AFB staining. Altogether, our results suggest that the tuberculosis CX-test accurately detects drug susceptible tuberculosis in low-income and high tuberculosis-burden settings.

A previous study of 702 patients at a HIV and tuberculosis treatment clinic in Lilongwe found a low (1.4%) prevalence of drug-resistant tuberculosis, with only 10 cases of culture-confirmed isoniazid resistance, and one case (0.1%) each for rifampicin resistance and MDR- tuberculosis.^20^ Our results also confirmed the lack of drug-resistant tuberculosis among participants in this study and thus we are unable to report on the tuberculosis CX-test’s ability to identify drug-resistant *M. tuberculosis.* Of a subset of positive samples (10%) sent for the GeneXpert MTB/RIF^®^ testing, the tuberculosis CX-test was in agreement with the GeneXpert MTB/RIF^®^, in that the samples were susceptible to rifampicin. None of the participants who stated that they had taken first-line anti-tuberculosis drugs for susceptible tuberculosis had failed therapy, further suggesting the absence of clinically-significant drug-resistant tuberculosis.

Although there are several commercially-available molecular tuberculosis diagnostic tests on the market,^21^ including the GeneXpert MTB/RIF^®^, which has exceptional sensitivity, specificity, and minimal time to results, GeneXpert tests are currently cost prohibitive in many low-income countries, and especially in countries with a high tuberculosis burden. As a result, the long-term implementation of such diagnostic tests requires ongoing financial support from governments or nongovernmental organisations such as the WHO, the United States Agency for International Development, and the World Bank, among others.

### Limitations

Of the 96 valid tests carried out in this study, three (3%) showed late growth in the rifampicin quadrant several days after growth was detected in the detection quadrant; results from the GeneXpert MTB/RIF^®^ test indicated that the three samples were all drug susceptible. There are several possible explanations for the late growth in the tuberculosis CX-test in the drug-containing quadrants days after growth was observed in the detection quadrant. This may be as a result of either a spontaneous mutation allowing bacteria to grow in the drug-containing quadrant, or could be due to breakdown of drugs after a certain period of time. Alternatively, the drug concentration in the quadrants with late growth could be suboptimal (thereby giving a bacteriostatic effect instead of a bactericidal effect), or samples could contain bacteria that are heteroresistant. Indeed, tuberculosis patients may harbour both drug susceptible and -resistant *M. tuberculosis* strains. Although these are minor possibilities, these point out the importance of reading drug resistance results on the same day that the growth is observed in the detection quadrant (drug susceptible) of the tuberculosis CX-test. In the case that growth is observed in the drug-containing quadrants within seven days after being observed in the detection quadrant, the late growth will need to be further confirmed by GeneXpert MTB/RIF^®^ testing or other molecular techniques to rule out heteroresistance or a false negative result.

Other limitations include the small sample size and that the same personnel tabulated both the AFB smear and tuberculosis CX-test. Findings presented herein showed good correlation to AFB smear results; however, AFB microscopy (the only method used in the clinic where this study was performed) has been shown to have poor sensitivity or specificity when compared to either GeneXpert MTB/RIF^®^ or liquid culture (Mycobacteria Growth Indicator Tube [MGIT]) tests. Importantly, the time to detection of a positive tuberculosis CX-test was between 7 and 14 days, which is at least comparable to the time to detection for positive MGIT results for *M. tuberculosis* cultures. Finally, a correlation of smear grades to time to detection of the tuberculosis CX-test would also be useful in providing further comparators of performance.

### Recommendations

To determine the accuracy of diagnosis of tuberculosis drug resistance, the tuberculosis CX-test needs to be tested in countries with a high prevalence of drug resistance, where it can be compared to culture-confirmed drug susceptibility testing and GeneXpert MTB/RIF^®^.

### Conclusions

A tuberculosis test that is easy-to-use and inexpensive does not require expensive equipment and highly-specialised staff, and is relatively quick to detect drug resistance (~14 days vs. 86 days), is currently in demand in many low-resource, high tuberculosis-burden countries. The tuberculosis CX-test, with a current production cost of $2.00 USD, could be the necessary tool to fill this gap. While our findings support the accuracy of the tuberculosis CX-test in detecting active pulmonary tuberculosis, a larger study focusing on the ability to detect drug susceptibility is needed before this test could be evaluated for implementation as a front-line drug susceptibility test. A comparison of the tuberculosis CX-test to either GeneXpert MTB/RIF^®^ or MGIT culture would also be useful and is recommended to show the CX-test as an alternative to either of these platforms. In a setting of low drug resistance, this test is less useful than AFB smear, which is cheaper and less labour intensive. Thus, we hypothesise that the greatest utility for the tuberculosis CX-test is an environment with higher prevalence of MDR or XDR tuberculosis.
